# Bone marrow mesenchymal stem cell-derived exosomal microRNA-335 alleviates vascular calcification by targeting SP1

**DOI:** 10.3389/fcvm.2025.1572045

**Published:** 2025-09-26

**Authors:** Yaodong Li, Chuanzhen Chen, Jingbo Kong, Junjie Huang, Liguo Liu, Cunfa Liu, Bing Dai, Mei Huang

**Affiliations:** ^1^ Department of Vascular Surgery, Tianjin Hospital, Tianjin, China; ^2^ Tongji Hospital Affiliated to Huazhong University of Science and Technology, Wuhan, Hubei, China

**Keywords:** BMSCs, exosomes, miR-335, SP1, RUNX2, vascular calcification, VSMCs

## Abstract

**Background:**

Vascular calcification (VC) is a critical pathological characteristic of cardiovascular diseases like atherosclerosis, frequently linked to phenotypic alterations in vascular smooth muscle cells (VSMCs) and the activation of bone-forming genes. Exosomes derived from bone marrow mesenchymal stem cells (BMSCs) have been shown to significantly attenuate VSMC calcification.

**Methods:**

To investigate whether BMSC-derived exosomes mitigate VSMC calcification through microRNAs (miRNAs) regulation, we developed an *in vitro* model using β-glycerophosphate-induced calcification and an *in vivo* model using vitamin D3-induced calcification. Exosomes were extracted from BMSC culture media via ultracentrifugation and analyzed using transmission electron microscopy and particle size distribution assays.

**Results:**

Functional and phenotypic assessments revealed that BMSC-derived exosomes markedly reduced VSMC calcification. RT-qPCR analysis further indicated that BMSC-derived exosomes regulate VSMC calcification by modulating rno-miR-335 (miR-335). The miR-335 mimic notably suppressed the expression of the osteogenic regulator RUNX2 in VSMCs. Dual-luciferase reporter assays demonstrated that SP1 is a direct target of miR-335. Exosomal miR-335 inhibited SP1 expression, resulting in reduced mRNA and protein levels of RUNX2. *In vivo* studies confirmed that agomiR-335 treatment significantly lowered SP1 levels in the aorta of male SD rats, alleviating vitamin D3-induced VC.

**Conclusion:**

This study highlights that BMSC-derived exosomes regulate VC via the miR-335/SP1 axis, offering novel molecular targets for treating VC.

## Introduction

Vascular calcification (VC) is a progressive pathological disorder that worsens with age and is further aggravated by chronic conditions such as hypertension, chronic kidney disease, and diabetes mellitus (1). This disease primarily affects the intima and media of large arteries, where vascular smooth muscle cells (VSMCs) undergo a phenotypic transition to an osteoblast-like state under pathological conditions, characterized by reduced expression of VSMCs-specific markers (2–5). Despite advances in understanding VC pathogenesis, the molecular mechanisms driving VSMCs calcification remain unclear, hindering the development of effective therapeutic strategies to halt disease progression.

Emerging evidence highlights the critical role of cell-cell interactions in VC development, particularly between bone marrow mesenchymal stem cells (BMSCs) and VSMCs (6). BMSCs, known for their multidirectional differentiation potential, secrete exosomes that act as key mediators of tissue repair in traumatic brain injury and myocardial infarction models (7, 8). Notably, BMSC-derived exosomes have recently been implicated in regulating VSMCs phenotypic switching and calcification (9). These extracellular vesicles exert their biological effects by transferring cargo molecules, including microRNAs (miRNAs), which orchestrate intercellular communication between BMSCs and VSMCs (10, 11). Among these miRNAs, rno-miR-335 (miR-335) has garnered increasing attention for its dual regulatory roles in osteogenic differentiation and vascular pathology, including cell differentiation, apoptosis, and tissue regeneration. While miR-335 promotes osteogenesis by targeting RUNX2, a master transcription factor for bone development (12–14), paradoxically, it appears to suppress VSMC calcification in atherosclerotic conditions (15, 16). This functional dichotomy suggests context-dependent roles of miR-335 in mineral metabolism, warranting further investigation.

Concurrently, the transcription factor SP1 has emerged as a critical regulator of VC pathogenesis (17, 18). SP1 promotes VSMCs osteogenic transdifferentiation by activating osteogenic gene programs, such as RUNX2, thereby accelerating calcium deposition in vascular walls (19–21). Intriguingly, miR-335 can directly target and inhibit SP1 to improve the functional recovery after spinal cord injury, promote diabetic wound healing and inhibit the apoptosis and invasion of lung cancer cells (22–24). Building on these findings, we hypothesize that miR-335-mediated suppression of SP1 may represent a novel regulator*y* axis in VC development.

This study aims to elucidate the functional role of BMSC-derived exosomal miR-335 in VC and its underlying mechanism through SP1 signaling. By dissecting the miR-335/SP1 interaction axis, our findings may provide new molecular targets for therapeutic interventions against VC.

## Materials and methods

### Cell acquisition and culture

In order to study the biological characteristics of VSMCs and BMSCs, the A7r5 cell line was sourced from the Chinese Academy of Sciences. VSMCs were cultured in DMEM (Thermo Fisher Scientific, USA), enriched with 10% FBS (Gibco, Australia) and 1% penicillin/streptomycin (Beyotime Biotechnology, China), under conditions of 37°C and 5% CO₂. BMSCs were isolated by extracting from the bone marrow of 3-month-old SD rats. The femur and iliac bones of the rats were removed using aseptic techniques, cut into small pieces, and washed in PBS to remove blood impurities. BMSCs were obtained by adding PBS to the bone cavity and gently rinsing. The collected cell suspension was filtered through a 40 µm mesh to remove large tissues, and then centrifuged (1,200 rpm, 5 minutes) to remove red blood cell sediments. The obtained bone marrow cell suspension was inoculated into DMEM medium containing 10% exosome-depleted FBS and 1% penicillin/streptomycin at 37°C, 5% CO₂. The cells were grown to 80% confluence and then passaged. BMSCs were identified by flow cytometry. We used the following markers to identify the phenotype of BMSCs: CD34 (1:100, Cyagen Biosciences), CD44 (1:100, Cyagen Biosciences), CD45 (1:100, Cyagen Biosciences), CD90 (1:100, Cyagen Biosciences). All antibodies were used with corresponding fluorescent-labeled secondary antibodies. Flow cytometry (BD FACSCalibur) was used for data collection and analysis, and the positive rate of cell surface markers was used to determine the purity of BMSCs. To ensure the reproducibility and reliability of the experiment, all cells were cultured under the same conditions, ensuring that the components of the culture medium and environmental factors supported optimal cell growth.

### Induction of calcification in VSMCs and interventions

In this study, VSMCs were cultured under specific conditions to investigate their response to factors promoting VC. The culture medium used for the VSMCs was DMEM, fortified with 10% FBS and 1% penicillin/streptomycin. When the confluence of cell growth reaches 80%, to avoid the influence of exosomes in the serum, we changed the medium which consisted of 10 mM β-glycerophosphate, 50 μg/ml ascorbic acid, and 10⁻⁸ mol/L dexamethasone, as well as DMEM medium containing 10% exosome-depleted FBS and 1% penicillin/streptomycin culture medium to induce VC. The medium was changed every 3 days. VSMCs were treated with the calcifying media for a period of 14 days. This protocol follows well-established methods reported in the literature (25), ensuring consistency with previous studies while providing a suitable experimental setup for studying the calcification process. In the Exos group, we added BMSC-derived exosomes at a concentration of 100 µg/ml into medium at the 6th day during the period of induced calcification (26). The control (Ctrl) group added an equal amount of PBS. Mimic-miR-335 (miR10000575-1-5) and mimic-Ctrl (miR1N0000001-1-5) purchased from RiboBio Co., Ltd, China. were carried out in VSMCs in six plates using Lipofectamine™ 3,000 (Thermo Fisher Scientific, USA) based on the manufacturer's instructions. Mimics were added to the medium at the 6th day during the period of induced calcification in mimic-control (mimic-Ctrl) group and mimic-miR-335 group to verify the effect of miR-335 in the process of inducing calcification. The culture of cells will be stopped after the completion of calcification induction. All the experiments were conducted separately for a minimum of three times.

### Isolation, identification and secretion of exosomes

After the culture period, the medium was subjected to centrifugation at 1,000 rpm for 5 minutes to eliminate intact cells, followed by a higher speed spin at 10,000 rpm for 30 minutes to separate larger vesicles. For exosome isolation, the supernatant underwent ultracentrifugation at 110,000 × g for 90 minutes at 4°C, a standard procedure to concentrate the exosome fraction. After ultracentrifugation, the exosome pellet was rinsed with phosphate-buffered saline (PBS) to eliminate any remaining contaminants before further analysis. To verify the presence and purity of exosomes, the samples were characterized using both Western blot and immunofluorescence techniques. Specific markers TSG101, were employed in these assays as reliable indicators for exosome identification, confirming the successful isolation and characterization of the exosomes derived from BMSCs. The exosomes were labeled with PKH26 (4 μM, Sigma-Aldrich, USA) following the manufacturer's protocol, with the final exosome concentration adjusted to 50 mg/L as indirectly quantified by measuring protein content with BCA assay (27). After 5-minute incubation, the labeled exosomes were co-cultured with VSMCs, and monitored at 6, 12, and 24 hours using confocal microscopy (Leica TCS SP8, Germany) to further demonstrate the dynamics of exosome internalization.

### RT-qPCR experiments

Total RNA was extracted from the samples using NucleoZOL (Macherey-Nagel, Germany). For the expression of miRNA, we used a kit specifically for miRNA extraction (MirVana miRNA Isolation Kit, Thermo Fisher Scientific, USA) to ensure efficient extraction of miRNA. To synthesize complementary DNA (cDNA) from RNA, we used a reverse transcription kit specifically for miRNA (TaqMan MicroRNA Reverse Transcription Kit, Thermo Fisher Scientific, USA) to ensure efficient reverse transcription of miRNA. Afterwards, PCR was performed using PowerTrack™ SYBR Green Master Mix for qPCR (Thermo Fisher Scientific, USA), which is known to have high sensitivity and reliability and is able to detect specific sequences. The primers were synthesized by RiboBio Co., Ltd, China. The reaction included 40 amplification cycles. The primer sequences used for qPCR are as follows:

#### SP1

Forward: 5'-ACCTGGCGGTGATGGAAT-3’

Reverse: 5'-GGTGGGTCTTGATATGCTTTG-3’

#### RUNX2

Forward: 5'-ATGGCCGGGAATGATGAG-3’

Reverse: 5'-TGTGAAGACCGTTATGGTCAAAGTG-3’

#### β-actin

Forward: 5'-GGAGATTACTGCCCTGGCTCCTA-3’

Reverse: 5'-GACTCATCGTACTCCTGCTTGCTG-3’

#### rno-miR-335

Sequence: TCAAGAGCAATAACGAAAAATGTAA

#### U6

Forward: 5′-CTCGTCGGCAGCAA-3′

Reverse: 5′-AACGCTTCACGA ATTTTGCGT-3′

### ALP staining

ALP staining was used to evaluate the osteogenic differentiation ability of cells. Cells were stained in ALP staining solution (Sigma-Aldrich, ALP staining kit) for 30 minutes. After staining, cells were washed three times with PBS and the appearance of ALP-positive areas was observed under a microscope. ALP-positive areas indicate calcification-related osteogenic activity. The detailed steps of the staining process include the preparation of the solution, the staining time, and the observation criteria under a microscope and the results were analyzed by Image J.

### Alizarin red S staining

Alizarin Red S staining was used to evaluate the calcification deposition of cells. After calcification induction culture, cells were stained with Alizarin Red S solution (Sigma-Aldrich) for 10 minutes and then washed with PBS until there was no unbound dye. Finally, the dye was dissolved in 80% ethanol and quantitatively analyzed by spectrophotometer (OD 405 nm) to evaluate the degree of calcification deposition. The area of calcification was quantified by Image J and the differences between different experimental groups were compared.

### ALP activity assay

ALP activity assay was used to quantitatively analyze the osteogenic activity. After the cultured VSMCs and aorta tissue were treated under calcification induction conditions, the ALP activity assay kit (Sigma-Aldrich) was used for detection. According to the instructions of the kit, ALP activity was determined by colorimetry, and a standard curve was used to quantify the ALP enzyme activity in each sample. In the experiment, the cell lysate was collected after high-speed centrifugation and the supernatant after centrifugation of aorta tissue grinding homogenization. Both were incubated with ALP substrate at 37°C for 30 minutes, and the optical density value was determined by a microplate reader at a wavelength of 405 nm after the reaction was terminated. Each group of samples was repeated three times, and the data obtained were compared by statistical analysis.

### Western blotting

Cellular/tissue specimens or purified exosomes were homogenized in ice-cold RIPA lysis buffer (Solarbio, China) containing protease and phosphatase inhibitors. After 30-min incubation on ice, the lysates were centrifuged at 12,000 × g for 15 min at 4°C to obtain supernatants. Protein quantification was performed using BCA Protein Assay Kit (Beyotime Biotechnology, China) according to the manufacturer's instructions. Equal quantities of 15 μg protein samples were initially mixed with a loading buffer and subjected to electrophoresis using a 12% SDS-PAGE. After electrophoresis, the proteins were transferred to a PVDF membrane, known for its excellent protein binding capacity and suitability for Western blot analysis. To block non-specific interactions, with 5% non-fat milk for one hour. Following the blocking step, the membrane was incubated overnight with primary antibodies targeting the proteins of interest: Runx2 (1:1,000, Abcam, USA), GAPDH (1:2,000, Proteintech, China), SP1 (1:1,000, Proteintech, China) and TSG101 (1:1,000, Proteintech, China). The next day, after washing the membrane with TBST, the membrane was treated with a horseradish peroxidase (HRP)-linked secondary antibody for one hour. Detection was performed using the FluorChem FC3 imaging system (ProteinSimple, USA) with the ECL chemiluminescent substrate kit. The protein expression was then quantified using Image J.

### Immunofluorescence staining

VSMCs were first treated with a 4% paraformaldehyde solution for 10 minutes to preserve the cell structure and integrity. To allow the antibodies to enter the cells, 0.1% Triton X-100 (Solarbio, China) was used. The cells were subsequently exposed to primary antibodies α-SMA (1:1,000, Proteintech, China) overnight at 4°C. On the following day, the cells were exposed to secondary antibodies conjugated with fluorescent tags for one hour to facilitate the detection of primary antibody binding. To stain the nuclei, the cells were treated with DAPI for 10 minutes, a commonly used DNA-binding fluorescent dye. Lastly, the fluorescently labeled cells were visualized under a fluorescence microscope (IX71-olympus, Japan) to evaluate the distribution and expression of the target proteins, with the intensity of fluorescence indicating protein abundance and localization within the cells.

### Transwell co-culture system

To investigate the influence of BMSC-derived exosomes on VSMC activity, a Transwell co-culture system (Corning-3450, NY, USA) was employed, where BMSCs (1 × 10⁵ cells/well) were seeded in the lower chamber and VSMCs (5 × 10⁴ cells/well) in the upper chamber. BMSCs were cultured in DMEM supplemented with 10% exosome-depleted FBS, 1% penicillin/streptomycin, with or without 10 μM GW4869 (Sigma-Aldrich, #G5796) in GW4869 + group or GW4869- group (28). GW4869 was added 48 hours prior to co-culture during co-culture to inhibit exosome biogenesis via the ceramide pathway, and removed by replacing the medium. VSMCs (5 × 10⁴ cells/well) were seeded in the upper chamber in osteogenic induction medium (DMEM supplemented with 10 mM β-glycerophosphate, 50 μg/ml ascorbic acid, and 10⁻⁸ mol/L dexamethasone), while BMSCs (1 × 10⁵ cells/well) in the lower chamber were maintained in standard growth medium (DMEM with 10% exosome-depleted FBS and 1% penicillin/streptomycin). After 48 hours of co-culture, VSMCs were harvested for analysis of gene expression and alkaline phosphatase (ALP) activity. This design ensures that observed effects on VSMCs are primarily mediated by BMSC-derived exosomes, as GW4869 specifically blocks exosome production without interfering with other soluble factors (29).

### Dual-luciferase reporter assay

The online public databases RNA22 (https://cm.jefferson.edu/rna22) was used to identify possible mRNA binding sites that were regulated by rno-miR-335. After examining the intersection analysis of the results, we considered that SP1 was a significant binding candidate. To further determine the regulatory relationship between miR-335 and SP1, we performed a dual-luciferase reporter gene assay in HEK293 T cells (ATCC® CRL-3216™). In this experiment, we transfected wild-type SP1 3′-UTR plasmid (H19123) or mutant SP1 3′-UTR plasmid (H19124) and miR-335 mimic or the relative control in HEK293 T cells. A Dual-Luciferase Assay System (Promega Corporation, USA) was used to analyze firefly luciferase activity using Lipofectamine™ 3,000 as a transfection reagent, according to the manufacturer's directions.

### *In vivo* VC model

For the *in vivo* model, 3-month-old male SD rats (SPF grade, weighing 150–200 g) were obtained from the Guangxi Medical University Animal Research Facility. The rats were housed in a controlled environment with a temperature of 22 ± 2°C, humidity of 50%–60%, and a 12-hour light/dark cycle. Animals were provided with standard laboratory chow and sterile water *ad libitum*. Housing density and enrichment were maintained according to animal welfare guidelines, including the provision of environmental enrichment such as toys and nesting materials to reduce stress.

The VC rats were induced through subcutaneous administration of 300,000 IU/kg vitamin D3 once daily for 14 consecutive days. VC rats were divided randomly into agomiR-335 group and control group (Ctrl group) equally. In the agomiR-335 group, rats received agomiR-335 (5 nmol, miR40000575-4-5) intravenously every three days, starting on the first day of vitamin D3 treatment. In the Ctrl group, rats were administered agomiR-NC (5 nmol, miR4N0000001-4-5) in the same way and frequency. All the agomiRs were purchased from RiboBio Co., Ltd, China, and carried out under the manufacturer's instructions. The Negative control rats (NC group) were induced equal amounts of saline by the same route for the same duration. All interventions were carried out under 150 mg/kg phenobarbital anesthesia to minimize animal distress, with dosages adjusted according to animal weight.

At the end of the 14-day intervention, 150 mg/kg phenobarbital was used for anesthesia and euthanasia was performed by cervical dislocation after anesthesia according to AVMA guidelines. In addition, a mild anesthetic induction agent was combined during anesthesia to ensure a rapid onset of anesthesia. The thoracic aorta was carefully collected and processed for histological analysis. Aorta samples were fixed in paraformaldehyde, embedded in paraffin, and sectioned into 5 µm slices for subsequent Von Kossa and immunohistochemistry to evaluate VC. All experimental procedures adhered to the 3R principles (replacement, reduction, refinement) to minimize animal suffering. The study was approved by the Ethics Committee of Tianjin Hospital (Approval No. 2024-Yi-Lun-Shen-219).

### Von Kossa staining and immunohistochemistry staining of the aorta

To assess VC in rat aortic tissues, paraffin-embedded arterial cross-sections were subjected to Von Kossa staining (IW3014, IHC World) following the same procedure described by the manufacturer. This histochemical method specifically identifies calcium phosphate deposits through a silver nitrate reaction, wherein silver ions replace calcium in mineralized areas and are subsequently reduced to metallic silver by UV light exposure, resulting in black-brown precipitates (30). For quantification, three non-consecutive sections per animal were imaged under bright-field microscopy (×200 magnification). Calcified areas were defined as regions with positive black staining and quantified using Image J. A standardized thresholding protocol was applied to all images to differentiate calcified areas from background, with results expressed as the percentage of calcified area relative to the total vascular cross-sectional area.

For immunohistochemical staining of rat thoracic aorta sections, after deparaffinization in xylene and rehydration through a graded ethanol series (100%, 95%, 80%), antigen retrieval was performed by microwave heating in 10 mM citrate buffer (pH 6.0) for 15 min. Sections were then blocked with 5% fetal serum in PBS containing 0.3% Triton X-100 for 1 h at room temperature to reduce nonspecific binding. Primary antibodies against SP1 (1:500, Proteintech, China) and RUNX2 (1:500, Abcam, USA) were diluted in antibody diluent (A1800, Solarbio) and applied to separate sections overnight at 4°C. Then, sections were incubated with corresponding secondary antibodies for 1 h at room temperature and treated with a diaminobenzidine (DAB) kit according to the manufacturer's guidelines. Cell nuclei were stained with hematoxylin, and then photographed using a light microscope. For quantification, three non-overlapping fields per section were analyzed. Positively stained areas were analyzed using Image J.

### Statistical analysis

Statistical analysis was performed using SPSS software (version 26.0). All data were expressed as mean ± standard deviation (SD). Comparisons between multiple groups were conducted using one-way analysis of variance (ANOVA), followed by Tukey's *post hoc* test for pairwise comparisons. For non-normally distributed data or when homogeneity of variance was not met, Kruskal–Wallis tests were applied. For all analyses, a *p*-value < 0.05 was considered statistically significant (*p < 0.05, **p < 0.01, ***p < 0.001). Graphs were prepared using GraphPad Prism software (version 9.0). All experiments were performed in triplicate, and *n* represents the number of biological replicates per group.

## Results

### BMSC-derived exosomes attenuate VSMC calcification

**Figure 1:**
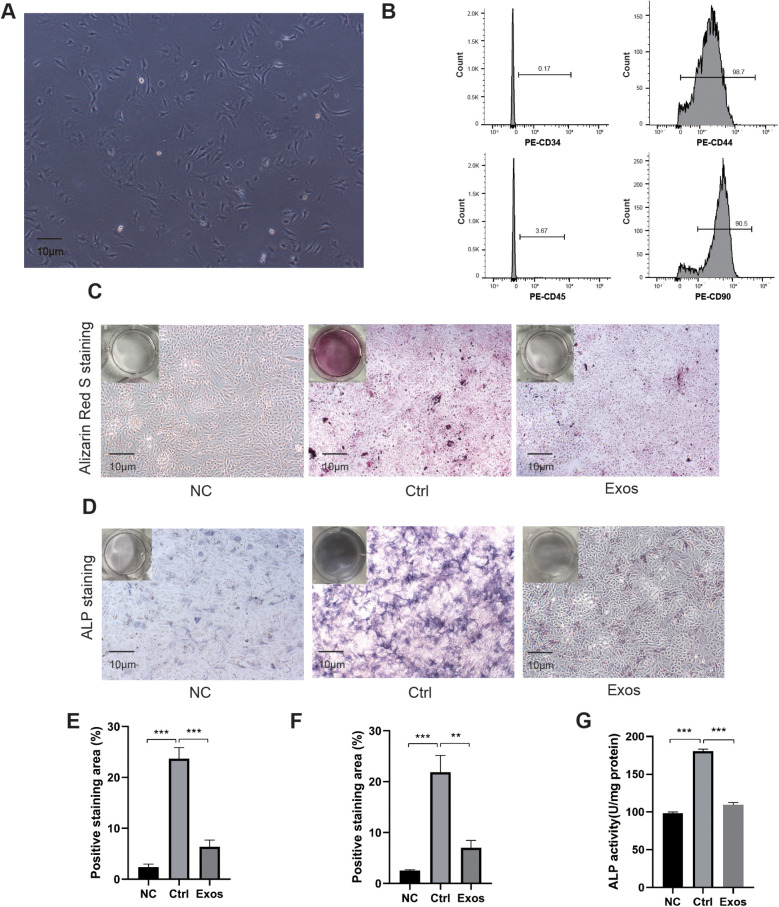
Exosomes secreted by BMSCs attenuate VSMC calcification. **(A)** Optical identification of BMSCs. **(B)** Flow cytometry identification of BMSCs. **(C)** Alizarin Red S staining of cultured VSMCs. **(D)** Alkaline phosphatase staining of VSMCs cultured in a six-well plate, photographed under optical microscopy. **(E)** Analysis of Alizarin Red S positive staining areas using Image J software (*n* = 3). **(F)** Analysis of alkaline phosphatase positive staining areas using Image J software (*n* = 3). **(G)** Alkaline phosphatase activity assay (*n* = 3).

We first identified BMSCs using optical microscopy and flow cytometry (Figures 1A,B). Experimental results showed that exosomes originating from BMSCs significantly reduced the calcification of VSMCs. Alizarin Red S staining (Figures 1C,E), alkaline phosphatase staining (Figures 1D,F) and alkaline phosphatase activity assay (Figure 1G) revealed that calcification in VSMCs treated with exosomes was significantly reduced. These results suggest that BMSC-derived exosomes effectively alleviate the calcification of VSMCs.

### Mechanism verification of BMSC-derived exosomes in attenuating VSMC calcification

**Figure 2:**
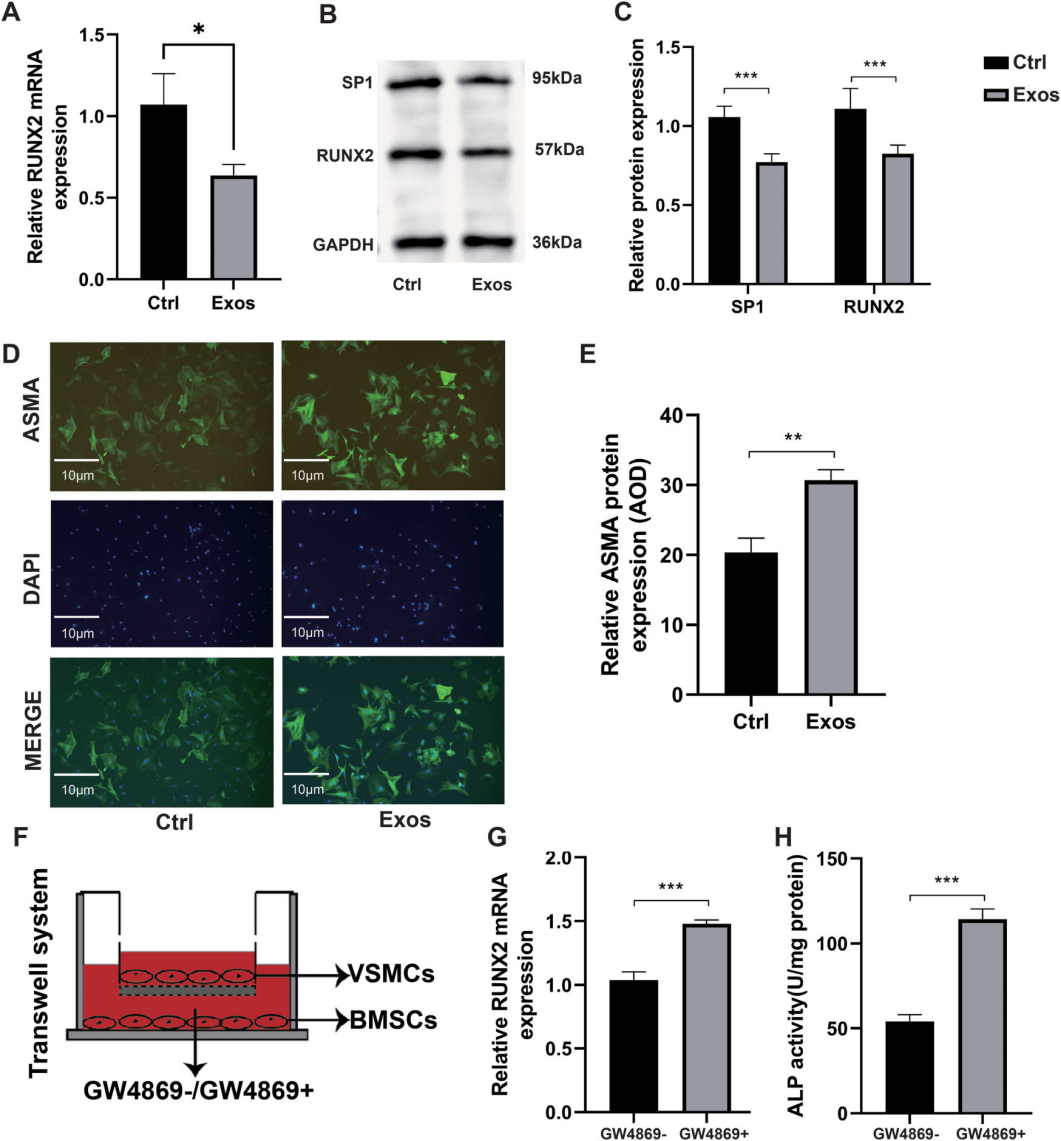
Mechanism verification of BMSC-derived exosomes in attenuating VSMC calcification. **(A–E)** VSMCs cultured in the media supplemented with PBS (control) or BMSC-derived exosomes at a concentration of 100 µg/ml (*n* = 3). **(A)** RUNX2 mRNA expression. **(B)** Western blot verification of SP1 and RUNX2. **(C)** Relative quantification of SP1 and RUNX2 protein expression. **(D)** Immunofluorescence measurement of ASMA using fluorescence microscopy. **(E)** Immunofluorescence analysis of ASMA and measurement of Average Optical Density (AOD). (**F–H**) BMSCs in the lower chamber were pre-treated for 48 hours with or without GW4869 in DMEM containing 10% exosome-depleted FBS. Subsequently, these BMSCs were co-cultured for 48 hours with VSMCs (upper chamber) maintained in osteogenic induction medium. This design ensured that exosome secretion from BMSCs was selectively inhibited prior to cellular crosstalk. **(F)** Schematic diagram of Transwell co-culture system. **(G)** Relative RUNX2 mRNA expression in co-culture (*n* = 3). **(H)** ALP activity assay in the co-culture system (*n* = 3).

Through RT-qPCR, we found that exosomes derived from BMSCs significantly inhibited the expression of RUNX2 mRNA (Figure 2A). Further WB (Figures 2B,C) also confirmed that exosomes suppressed RUNX2 protein expression. Additionally, the levels of α-smooth muscle actin (ASMA) were significantly increased in VSMCs treated with BMSC-derived exosomes (Figures 2D,E). We further validated the effect of exosomes secreted by BMSCs on VSMCs using a Transwell co-culture model (Figure 2F). To block exosome production and function, we employed GW4869 (10 µM), a known inhibitor of ceramide synthase and the classical exosome biogenesis pathway (29). The results showed that RUNX2 mRNA expression (Figure 2G) and ALP activity (Figure 2H) in VSMCs were increased compared with cells without GW4869 addition. This demonstrated that exosomes secreted by BMSCs can alleviate VSMC calcification.

### Characterization and uptake of BMSC-derived exosomal miR-335

**Figure 3:**
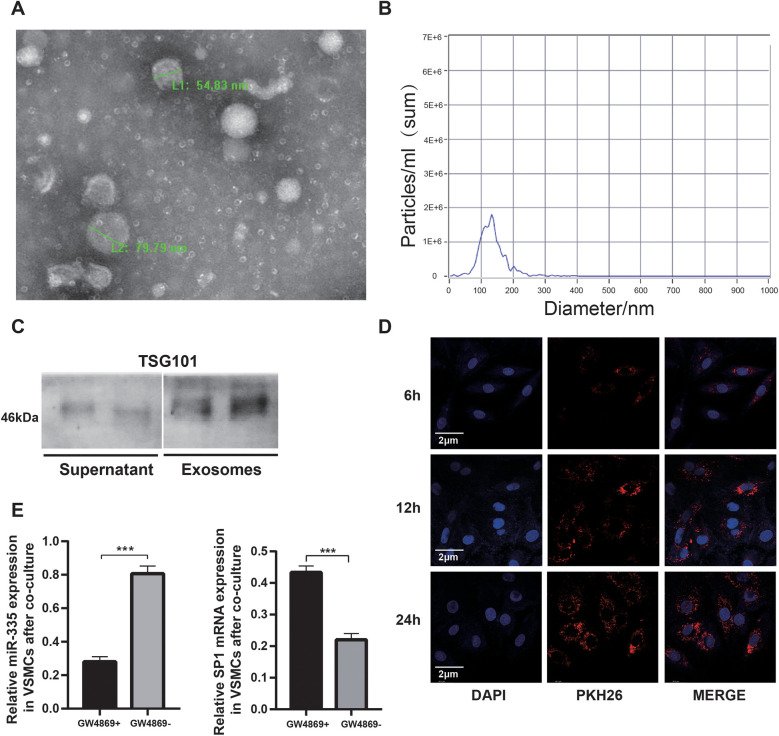
Characterization and uptake of BMSC-derived exosomal miR-335. **(A)** Representative TEM images showing cup-shaped exosomes with intact bilayer membranes. **(B)** Nanoparticle tracking analysis of exosomes. **(C)** Western blot analysis comparing the expression of TSG101 exosome-enriched vesicles with cell culture supernatant. **(D)** Confocal microscopy demonstrating PKH26-labeled exosomes (red) internalized by VSMCs (nuclei, DAPI, blue) at 6, 12, 24 hours, respectively. **(E)** qRT-PCR analysis analysis showing GW4869-dependent miR-335 downregulation resulted in increased SP1 mRNA levels in VSMCs (*n* = 3).

Transmission electron microscopy (TEM) analysis revealed that exosomes secreted by BMSCs exhibited characteristic cup-shaped or spherical morphology (Figure 3A). Nanoparticle tracking analysis demonstrated a predominant size distribution between 80 and 200 nm (Figure 3B), consistent with typical exosomal dimensions. Western blot analysis further confirmed the enrichment of exosomal marker TSG101 in the isolated vesicles (Figure 3C), validating successful exosome isolation from BMSC-conditioned medium. To investigate exosome-mediated intercellular communication, PKH26-labeled exosomes were co-cultured with VSMCs. Confocal microscopy showed that the number of exosomes internalized by VSMCs gradually increased with the extension of co-culture time (Figure 3D), thus establishing functional vesicle uptake. Mechanistic studies using the exosome secretion inhibitor GW4869 demonstrated significant downregulation of miR-335 in VSMCs following BMSCs co-culture (Figure 3E, left panel). This observation suggests that exosomal transport represents the primary route for miR-335 transfer between these cell types. Notably, qRT-PCR analysis revealed GW4869-dependent alterations in SP1 mRNA levels (Figure 3E, right panel), potentially attributable to exosome-delivered miR-335 mediating post-transcriptional SP1 mRNA destabilization via RNA-induced silencing complex (RISC) recruitment. This observation aligns with previously reported miRNA mechanisms that promote mRNA degradation (31).

### SP1 is a target gene of miR-335

**Figure 4:**
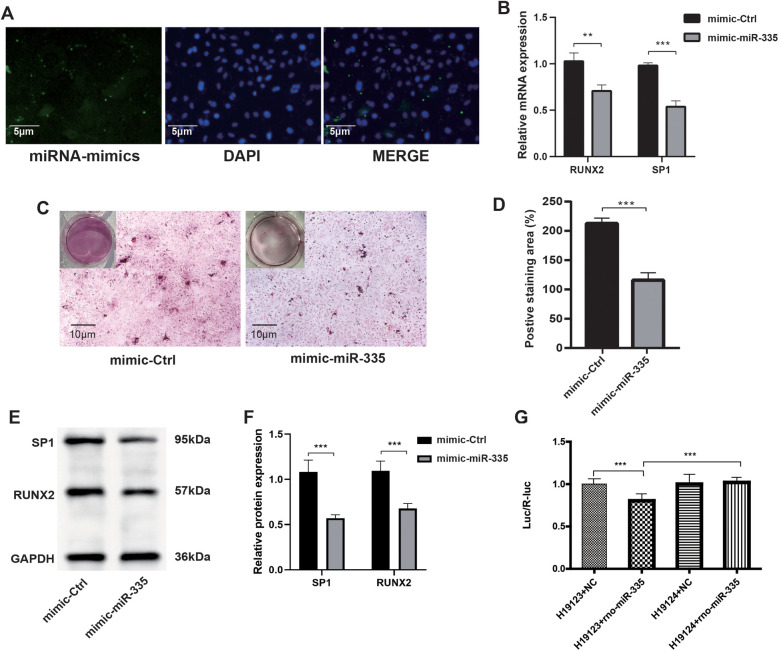
After the transfection of VSMCs with the miR-335 mimic, the expression of SP1 was suppressed, which in turn suppressed the calcification of VSMCs. **(A)** Fluorescence microscopy illustrating the transfection of miR-335 mimic. **(B)** Relative RUNX2 and SP1 mRNA expression (*n* = 3). **(C)** Alizarin Red S staining of cells. **(D)** The positive staining areas were measured using Image J software (*n* = 3). **(E)** Western blot validation of RUNX2 and SP1 proteins after miR-335 mimic transfection. **(F)** Relative quantification of RUNX2 and SP1 protein expression (*n* = 3). **(G)** Dual-luciferase reporter assay confirming SP1 is targeted by miR-335 (*n* = 6).

To confirm that miR-335 attenuated calcification in VSMCs, we transfected miR-335 mimics into VSMCs. Fluorescence microscopy showed that VSMCs successfully internalized the miR-335 mimic (Figure 4A). Mimic-miR-335 was shown to significantly reduce the calcification of the VSMCs by using Alizarin Red S staining (Figures 4C,D). Mechanistically, RT-qPCR showed miR-335 overexpression decreased SP1 and RUNX2 mRNA (Figure 4B). Western blot analysis (Figures 4E,E) paralleled these findings at protein level. The coordinated downregulation suggests SP1 may mediate RUNX2 suppression, as SP1 is known to transcriptionally activate RUNX2 in VC. Dual-luciferase reporter assays in HEK293 T cells provided direct evidence: miR-335 mimic reduced Luc/R-luc ratio when co-transfected with H19123 group (wild-type SP1 3'UTR), but not H19124 group (mutant construct) (Figure 4G). This confirms miR-335 directly targets SP1 through 3'UTR binding.

### *In vivo* verification of miR-335 in alleviating VC

**Figure 5:**
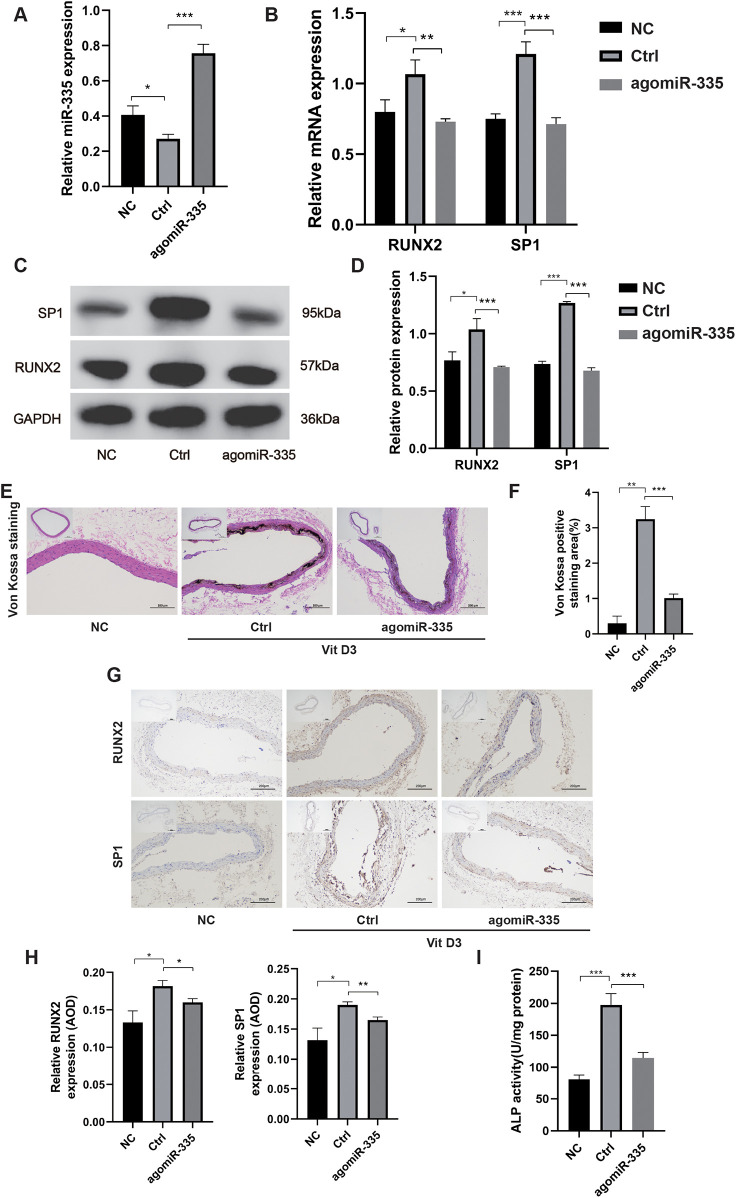
*in vivo* verification of miR-335 in alleviating VC (*n* = 3 per group). **(A)** MiR-335 expression in rat aorta. **(B)** Relative expression of RUNX2 and SP1 mRNA. **(C)** Western blot validation of RUNX2 and SP1 protein expression. **(D)** Quantification of relative protein expression of RUNX2 and SP1. **(E)** Von Kossa staining of rat aorta. **(F)** Analysis of Von Kossa staining positive areas using ImageJ software. **(G)** Immunohistochemical detection of RUNX2 and SP1 protein expression. **(H)** Analysis of the average optical density (AOD) of RUNX2 and SP1 using ImageJ software. **(I)** Alkaline phosphatase activity assay of the aorta tissue. NC, negative control; Ctrl, agomiR control.

**Figure 6:**
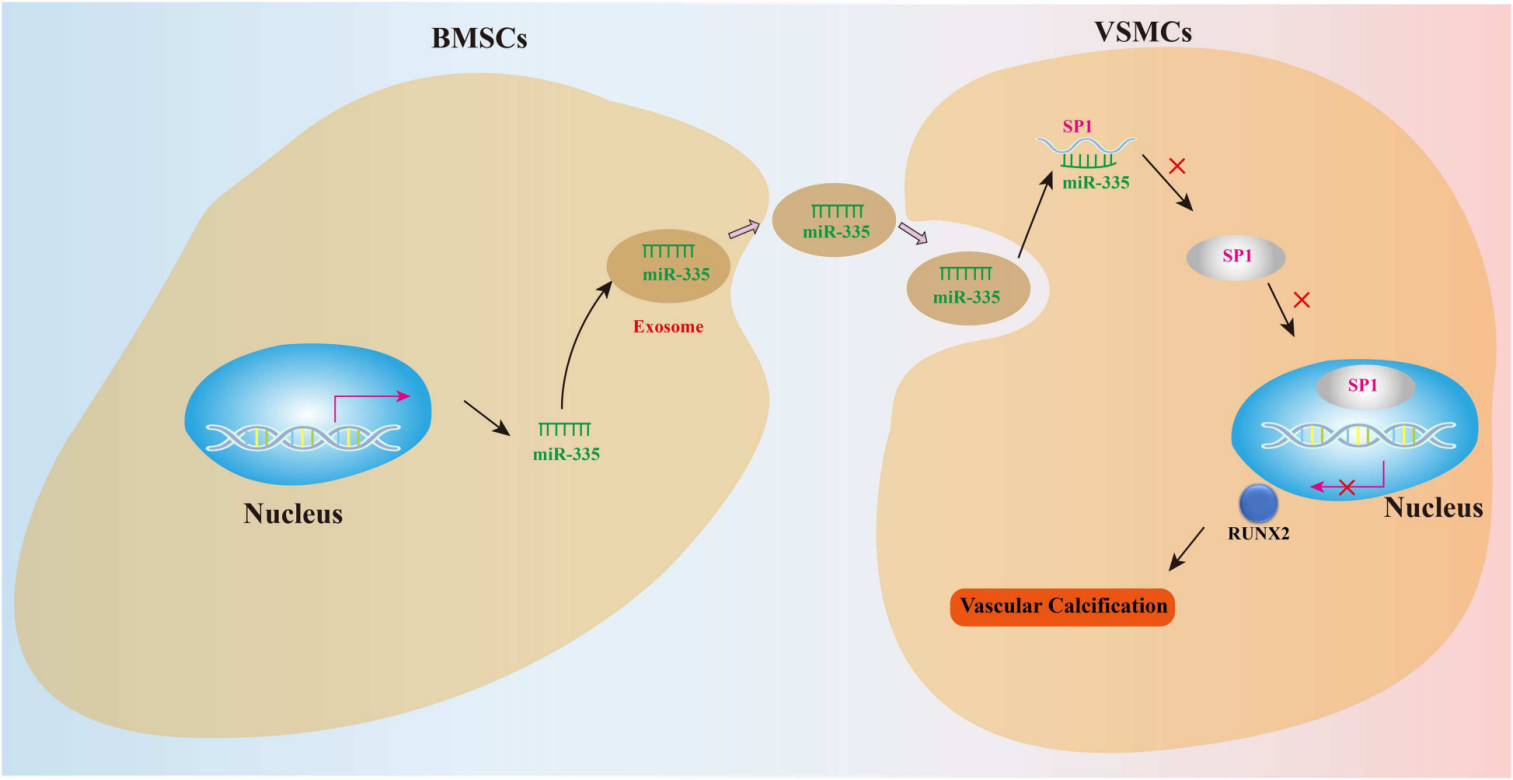
Mechanism diagram.

To further investigate the role of miR-335 in VC, we treated the rat VC model with either agomiR-335 (agomiR-335 group) or agomiR control (Ctrl group). The negative control (NC) group received equivalent volumes of saline administered identically in route and duration to the treatment groups without VC. Rat aorta extracted for RT-qPCR analysis showed that miR-335 expression in the Ctrl group was significantly lower compared to the NC and agomiR-335 group (Figure 5A). This means that the calcified environment will reduce the expression of miR-335 in aorta tissue, and the injection of agomiR-335 can increase the content of miR-335. In the Ctrl group, both mRNA (Figure 5B) and protein levels (Figures 5C,D) of RUNX2 and SP1 were markedly higher than agomiR-335 and NC group. Histological analysis via Von Kossa staining (Figures 5E,F) and immunohistochemical staining (Figures 5G,H) further confirmed that the establishment of the rat VC model was successful and agomiR-335 treatment effectively alleviated VC. The ALP activity in the agomiR-335 group was significantly lower than that in the Ctrl group, indicating that miR-335 alleviates VC by inhibiting ALP activity in arterial tissue (Figure 5I). These findings suggest that elevating miR-335 levels directly through agomiR-335 significantly mitigates vitamin D3-induced VC. Collectively, our results indicate that agomiR-335 can directly inhibit VSMC calcification and improve the pathological state of VC by targeting SP1, providing a promising therapeutic strategy independent of exosomal delivery systems (Figure 6).

## Discussion

VC is a hallmark pathological feature of atherosclerosis, and is typically associated with the phenotypic shift of VSMCs, calcium salt deposition, and the activation of osteogenic genes (32–35). Therefore, developing targeted therapeutic strategies for VC remains a critical challenge. Our previous study demonstrated that curcumin inhibits VC through the exosomal miR-92b-3p/KLF4 axis in VSMCs (36). In this study, we demonstrated that miR-335, delivered via BMSC-derived exosomes, regulates its target gene SP1, and then significantly inhibits VC, highlighting the crucial role of exosomes in cell signaling.

BMSCs, a population of multipotent stromal cells with potent proliferative, multidifferentiation, and immunoregulatory capabilities, contribute significantly to tissue repair and disease pathogenesis through paracrine signaling and cellular interactions (37). Emerging evidence indicates that BMSC-derived exosomes delivering non-coding RNAs, particularly miRNAs, which remodel transcriptional and functional reprogramming via epigenetic modulation in recipient cells (38). In this study, we isolated BMSC-derived exosomes and demonstrated their efficient internalization by VSMCs through PKH26 fluorescent labeling-based tracking. Exosome treatment induced a marked upregulation of α-SMA accompanied by concomitant downregulation of SP1 and RUNX2 in VSMCs, suggesting their coordinated regulation orchestrates the suppression of osteogenic transdifferentiation. Intriguingly, the exosome-mediated attenuation of VC was significantly abolished by GW4869-induced inhibition of exosome biogenesis, providing functional validation of the exosome-dependent protective mechanism. Furthermore, the Transwell co-culture system showed increased RUNX2 expression and ALP activity in VSMCs in the upper chamber in GW4869 + group compared with that observed in the GW4869- group. Collectively, these findings suggest that BMSCs attenuate VC through derived exosomes.

The exosome-mediated transfer of miRNAs constitutes a central paradigm for EXO-driven tissue repair. MiR-335 is widely involved in the modulation of various biological functions and diseases. In tumors, miR-335 exhibits roles that suppress tumor growth and metastasis (39–42). At the same time, miR-335 is also essential for regulating cell differentiation, apoptosis, and angiogenesis. It significantly influences neural system development and the maturation of BMSCs (43–45). Strikingly, the role of miR-335 in VC is complex, involving both the inhibition of VSMCs phenotypic transition and the possible role in bone formation. Knockdown of DANCR can reduce the proliferation and migration ability of VSMCs by upregulating miR-335-5p, thereby alleviating the formation of atherosclerosis (46). Similarly, miR-335-5p significantly suppresses vascular media degeneration by inhibiting SP1 (15). Conversely, studies have also found that overexpression of miR-335-5p can promote bone formation and regeneration (44). In addition, ATF2-driven miR-335-5p to target ERK/1/2 expression plays a key role in the osteogenic differentiation of BMSCs, as the main component of the MAPK pathway (47). However, the effect of MAPK signaling on osteogenesis exhibits bidirectional regulation, functioning through nuanced modulation of differentiation dynamics rather than serving as a dominant anti-osteogenic pathway (48). According to current research, the bidirectional regulation of miR-335 potentially stems from the coordinated effects of gene targeting specificity, cellular heterogeneity, microenvironmental signaling integration and exosome-dependent mechanisms (44, 49, 50). Therefore, the mechanistic role of miR-335 in VC warrants further investigation. Our experiments demonstrate that co-culture with BMSCs significantly upregulates miR-335 expression in VSMCs, which suppressed VC.

Integrated bioinformatic prediction and dual-luciferase reporter assays identified SP1 as a putative target of miR-335-mediated regulation in this pathway. SP1 promotes VC by regulating the transcription of key genes, such as upregulating osteogenic-related genes like BMP2 and RUNX2, which drive the osteoblastic transformation of VSMCs and induce calcification. Additionally, SP1 regulates apoptosis-related genes, influencing VSMC apoptosis, a key initiating factor in VC. The acetylation status of SP1 enhances its ability to bind to gene promoters and activate transcription, promoting calcification, while deacetylated SP1 has the opposite effect. SP1 interacts with other proteins, such as Setd8, to regulate the transcription of genes like Mark4, which promotes VSMC apoptosis via the Akt pathway (21).These findings collectively establish SP1 as a central molecular switch governing VC pathogenesis. Collectively, this study demonstrates that BMSC-derived exosomes can regulate the miR-335 levels to downregulate the expression of SP1, which in turn reduces RUNX2 expression, significantly attenuating the calcification process *in vitro*. *in vivo*, administration of agomiR-335 also suppressed calcification via SP1.

Although we have drawn meaningful conclusions in this study, we also recognize that the current study has certain limitations. It is noteworthy that the inhibitory effect of BMSC-derived exosomal miR-335 in VC requires further experimental validation in subsequent studies. Despite evidence​ demonstrating that BMSC-derived exosomes at 100 μg/ml significantly inhibit VC (26), the dose-dependent mechanisms remain uncharacterized, necessitating further research incorporating systematic dose gradients is warranted to elucidate the dose-dependent mechanisms. Even though our experimental results indicate that miR-335 inhibits VSMC calcification by suppressing SP1 expression, the specific mechanism of SP1 in VC has not been directly verified. Future studies could confirm the signal pathway by using siRNA or CRISPR technology for SP1. RNA sequencing technology can provide us with more comprehensive molecular evidence, help further reveal the specific mechanism of BMSC-derived exosomes, and identify more possible regulatory factors.

## Conclusions

This study delineates the role of BMSC-derived exosomal miR-335/SP1/RUNX2 signaling axis in VC. We found that miR-335 in exosomes secreted by BMSCs, inhibits the expression of SP1 in VSMCs, leading to reduced RUNX2 activity and subsequently alleviating vascular mineralization. These findings provide new insights into the regulatory function of exosomes in intercellular communication and suggest their potential as therapeutic agents for cardiovascular diseases characterized by ectopic calcification.

## Data Availability

The datasets presented in this study can be found in online repositories. The names of the repository/repositories and accession number(s) can be found in the article/Supplementary Material.
